# Bacteria in milk from anterior and posterior mammary glands in sows affected and unaffected by postpartum dysgalactia syndrome (PPDS)

**DOI:** 10.1186/1751-0147-51-26

**Published:** 2009-06-22

**Authors:** Nicole Kemper, Imke Gerjets

**Affiliations:** 1 Institute of Animal Breeding and Husbandry, Christian-Albrechts-University Kiel, Olshausenstraße 40, D-24098 Kiel, Germany

## Abstract

**Background:**

The performance of piglet weight gain is strongly dependent on the sow's ability to meet the demand for adequate milk. Postparturient disorders, especially those subsumed under the term postpartum dysgalactia syndrome (PPDS), can alter or reduce the milk production sensitively, resulting in starving piglets. The aim of this study was to gather further information about the prevalence of different bacterial species in the anterior and posterior mammary glands of sows with respect to the clinical appearance of PPDS.

**Methods:**

In this study, the health status of 56 sows after farrowing was determined with special regard to mastitis and dysgalactia. Pooled milk samples from anterior and posterior glands were taken from both affected and non-affected animals and analysed bacteriologically for the presence of a wide spectrum of different pathogens.

**Results:**

Mainly *Escherichia coli*, staphylococci and streptococci were detected in high percentages but without significant differences in healthy and diseased animals and anterior and posterior glands. However, the large percentages of coliform bacteria suggested a transmission route via faecal contamination.

**Conclusion:**

In this study, the prevalence of different bacteria in anterior and posterior glands in PPDS positive and negative sows was analysed. No significant differences in bacteria of healthy and diseased sows were assessed. Therefore, the development of clinical PPDS and actual infection seems to be largely dependant on individual resistance in single sows.

## Background

In their first days of life, piglets are totally reliant on the sow for access to colostrum and milk. Every alteration in both milk yield and composition has highly sensitive impacts on weight gain and growth rate. Therefore, postparturient disorders, including dysgalactia in sows, are a very important disease complex economically [1]. They are reported world-wide, but subsumed under different terms depending on the geographical location. While mastitis, metritis and agalactia syndrome (MMA) [2,3] is the commonly used name in European countries, post-partum dysgalactia syndrome (PPDS or PDS) [4] has become widely accepted in English-speaking areas. At farm level, incidence is estimated to differ between 0.5% and 60% [5], with an average incidence of about 13% [6-11]. The syndrome is characterised by greatly reduced milk production within 12 to 48 hours post-partum that rapidly leads to piglet starvation. Even though metritis is often a part of the syndrome, mastitis is the central symptom, as shown by several studies [12-14]. The bacteria most frequently isolated from mastitis-affected sows belong to the class of coliforms [13-17]. Bacterial genera included in the class of coliforms are represented by *Escherichia*, *Enterobacter*, *Citrobacter*, and *Klebsiella*. Several successful infection experiments leading to mastitis in sows have demonstrated the predominant role of these organisms [13,18]. Mastitis is a clear pathological entity: infected glands typically show signs of inflammation such as severe oedema and skin congestion [4], and, with many glands involved, sows develop fever (>40.3°C) and lose their appetites [18-20]. Mastitis can be a local process, restricted to one or several glands, but can also affect all mammary complexes [4]. Pathological foci of mastitis were detected as well in anterior complexes as in posterior complexes, but severe changes were more present in the latter [20]. These findings were supported by Bostedt et al. [21] and Baer and Bilkei [22], showing that posterior glands were more prone to pathological changes compared with anterior ones. However, detailed investigations on the bacteriological findings in cranial and caudal mammary glands and the possible consequences have not yet been reported. Therefore, the presence of bacteria in mammary glands of different location was examined in this study with special regard to the clinical status of the sow post partum.

## Methods

### Animals

In our study, 56 primiparous or multiparous Large White and cross-bred sows, taking part in an experiment on injected temperature transponders and water intake [16], were investigated. The study took place in a time period of six months between December 2007 and May 2008. Sows were housed at the institute's research farm with 120 sows in total. They were managed in a three-week rhythm with a 28-day lactation period. At average, the sampled animals were in their 2,9^th ^parity. Sows were defined as PPDS-positive due to a combination of appropriate criteria: with a rectal temperature higher than 39.5°C within 12 to 24 hours post-partum, and in addition, detectable inflammation in the mammary gland, and/or diminished appetite and/or altered piglet behavior [1,12]. Inflammation was diagnosed by a veterinarian, observing the typical signs of inflammation, such as severe edema, hardening, and skin congestion. Altered piglet behavior was present when piglets show vigorous nursing efforts, decrease their attempts to nurse and their general activity, retreat to the warmest parts of the farrowing crate or lose weight.

### Bacteriology

After assessment of the PPDS status, the teats were cleaned with soap solution and disinfected with 70% isopropyl alcohol (Universal Hospital Supplies LTD, Enfield, UK). Milk samples from all animals were milked on transport swabs with Amies medium (Transwab, Medical Wire & Equipment, Corsham, UK) after oxytocin injection (30 I.E. i.m.). This oxytocin dosage was chosen after pre-tests on the minimum dosage followed by milk ejection, and also recommended by Morkoc [23]. Piglets were removed before the washing procedure, and five minutes after injection, milking was started. The first streams of secretion from each teat were discarded in order to 'wash out' bacteria in the distal end of the teat canal. Specimens were transported to the laboratory within two hours and pooled. Pooling was performed by incubating all samples of one animal from the anterior mammary complex, subsuming the first three pairs of pectoral glands, for enrichment in one tube (50 mL) with casobuillon (Roth GmbH&Co KG, Karlsruhe, Germany). Samples from the three to four following pairs of caudal glands were pooled likewise. Consequently, a sample pair consisting of pooled milk swabs from anterior and posterior glands had to be examined for each sow. In total, 112 pooled samples (56 from anterior and 56 from posterior glands) were analysed. After 24 hours of incubation at 37°C, bacteriological routine diagnostic procedures including selective enrichment and biochemical confirmation (API20E, bioMérieux, Nürtingen, Germany) took place as described previously for *Enterobacteriaceae*, especially *Escherichia coli *(*E. coli*), *Klebsiella *species (spp.), *Enterobacter *spp., *Citrobacter *spp., and *Salmonella *spp. [24]. *Staphylococci *were isolated via incubation in CSL-Bouillon (Roth GmbH&Co KG) (37°C, 24 h) and streaking out on Sheep Blood Agar (Oxoid GmbH, Wesel, Germany) (37°C, 24 h), followed by Gram-staining and Catalase-testing (Merck KGaA, Darmstadt, Germany) and biochemical identification (ID32Staph, bioMérieux). For *Streptococci*, incubation was performed in the same way and biochemical identification was done with API20Strep (bioMérieux).

### Statistical analysis

For statistical analysis, the Fisher's Exact Test was performed using the procedure PROC FREQ from the SAS statistical software (SAS, 2003). In general, a statistical significance level of p ≤ 0.05 was used.

## Results

**Figure 1:**
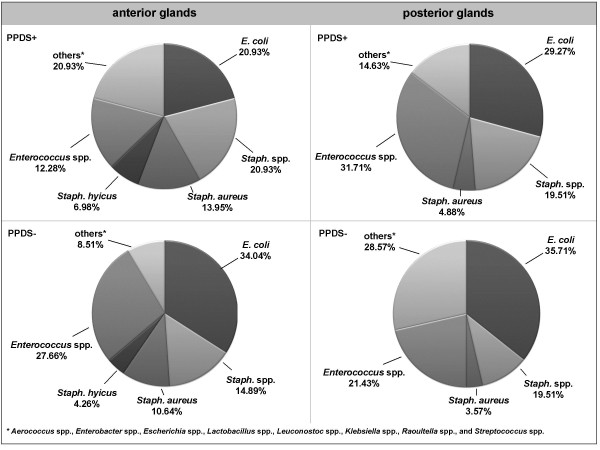
**Bacterial spectrum in samples from anterior and posterior glands from PPDS-affected (n = 27) and non-affected sows (n = 29)**.

According to the definition of PPDS affection, 27 sows (48.2%) out of the total number of 56 animals were positive. Consequently, 29 milk sample pairs from negative sows (51.8%) were available for investigation. From all sows, one or more different bacteria were isolated at least in one of the two pooled samples. In total, 159 bacteria isolates were detected. From PPDS-affected sows, 84 isolates were proven in anterior (43 isolates) and posterior samples (41 isolates), while 75 were present in anterior (47 isolates) and posterior (28 isolates) samples from non-affected sows. In both PPDS-affected and non-affected sows, no bacteria were detected in 18.5% (PPDS+) and 13.8% (PPDS-) of pooled samples from anterior glands and in 25.9% (PPDS+) and 34.5% (PPDS-) from posterior mammary glands (Table 1). This percentage was increased in the posterior glands in PPDS-negative sows. Furthermore, posterior mammary glands in negative sows showed the lowest variety of bacterial species. The isolated species belonged to the families *Enterobacteriaceae*, *Staphylococcaceae*, *Streptococcaceae*, and *Enterococcaceae *(Table 2). *Escherichia coli*, *Staphyloccocus *spp. and *Enterococcus *spp. were the predominant bacteria species. From the genus *Staphyloccocus *(*Staph*.), *Staph. aureus *and *Staph. hyicus *were considered in the following analysis at species level. Genera found only in very low numbers were subsumed as 'others', and included *Aerococcus *spp., *Enterobacter *spp., *Escherichia *spp., *Lactobacillus *spp., *Leuconostoc *spp., *Raoultella *spp., and *Streptococcus *spp.. Due to the possible importance as a pathogen, *Klebsiella *spp. were specially mentioned. After statistical analysis, only the percentages of *Staph. aureus *and *Staph. hyicus *differed significantly with regard to the mammary gland location (Table 3). This is, however, related to the low prevalence of these species (*Staph. aureus*: n = 14; *Staph. hyicus*: n = 6). The comparison of the bacterial occurrence in all samples of PPDS-affected and non-affected sows showed no significant influences of the PPDS status on the bacterial flora with the exception of *Staph*. spp., showing significant differences with respect to the PPDS status (Table 4). The distribution of the different bacteria in anterior and posterior pooled samples from PPDS-affected and non-affected sows is shown in Figure 1.

**Table 1:** Number of different bacteria species isolated in samples from PPDS-affected and non-affected sows

	Pooled samples from anterior mammary glands (first 3 pairs of cranial glands)						Pooled samples from posterior mammary glands (3 – 4 pairs of caudal glands)					
Number of isolated different bacteria species	0	1	2	3	4	5	0	1	2	3	4	5

In samples from PPDS+ sows (n = 27) (%)	18.5	29.7	25.9	25.9	0.0	0.0	25.9	25.9	25.9	18.5	0.0	3.8

In samples from PPDS- sows (n = 29) (%)	13.8	37.9	31.0	10.3	3.5	3.5	34.5	34.5	31.0	0.0	0.0	0.0

**Table 2:** Numbers of isolated bacteria in all samples (n = 112)

*Enterobacteriaceae*		*Staphylococcaceae*		*Streptococcaceae*		*Enterococcaceae*	
	n		n		n		n
*Enterobacter *sp.	2	Staph*. aureus	14	*Aerococcus urinae*	4	Ec*. avium	2
*Escherichia coli*	47	*Staph. capites*	1	*Aerococcus viridans*	3	*Ec. durans*	16
*Escherichia vulneris*	2	*Staph. chromogenes*	2	*Aerococcus *sp.	2	*Ec. faecalis*	3
*Klebsiella oxytoca*	1	*Staph. equorum*	1	*Lactococcus lactis*	2	*Ec. faecium*	9
*Klebsiella pneumoniae*	1	*Staph. hyicus*	6	Str*. bovis	1	*Ec*. sp.	9
*Raoultella ornithinolytica*	1	*Staph. lentus*	1	*Str. equinus*	1		
		*Staph. saprophyticus*	1	*Str. mitis*	2		
		*Staph. simulans*	14	*Str. mutans*	1		
		*Staph. warneri*	1	*Str*. sp.	5		
		*Staph*. sp.**	5				

*Staphylococcus (*Staph*.), *Streptococcus *(*Str*.), *Enterococcus *(*Ec*.)

** not further identified

**Table 3:** P-values of the effects 'PPDS-status' and 'mammary gland location' on the occurrence of different bacteria species in samples from PPDS-affected and non-affected sows (n = 112)

	PPDS status	Mammary gland location
*E. coli*	0.13	0.13

*Staph. aureus*	0.18	**0.02**

*Staph. hyicus*	0.33	**0.01**

*Staph*. spp.*	**0.04**	0.14

*Enterococcus *spp.	0.16	0.15

*Klebsiella *spp.	0.23	0.51

others*	0.18	0.19

significant effects (≤ 0.05) in bold

* all *Staph*. species but *Staph. aureus *and *Staph. hyicus*

** *Aerococcus *spp., *Enterobacter *spp., *Escherichia *spp., *Lactobacillus *spp., *Leuconostoc *spp., *Raoultella *spp., *Streptococcus *spp.

**Table 4:** Percentage of isolated bacteria species in the total number of samples (n = 112) from PPDS-affected and non-affected sows

	percentage in samples (n = 54) from PPDS+ sows (%)	percentage in samples (n = 58) from PPDS- sows (%)	p-values
*E. coli*	38.9	44.8	0.13

*Staph. aureus*	14.8	10.3	0.18

*Staph. hyicus*	11.1	10.3	0.32

*Staph*. spp.*	29.6	15.5	**0.04**

*Enterococcus *spp.	33.3	31.0	0.16

*Klebsiella *spp.	3.7	0.0	0.23

others**	20.4	17.2	0.18

significant effects (≤ 0.05) in bold

* all *Staph*. species but *Staph. aureus *and *Staph. hyicus*

** *Aerococcus *spp., *Enterobacter *spp., *Escherichia *spp., *Lactobacillus *spp., *Leuconostoc *spp., *Raoultella *spp., *Streptococcus *spp.

## Discussion

The aim of this study was to gather further information about the prevalence of different bacterial species in the anterior and posterior mammary glands of sows with respect to the clinical appearance of the PPDS syndrome. This clinical appearance is usually diagnosed via rectal temperature measuring post-partum [25] and sows are defined as 'PPDS-positive' when a certain threshold is exceeded. This threshold is defined rather coincidentally between 39.3°C and 40.5°C [3] and the use of it might be regarded as critical since physiological hyperthermia is often observed in sows, leading to misinterpretations [4,16]. To avoid confusion with this physiological temperature increase and to reduce the administration of antibiotics in non-affected animals, a threshold of 39.5°C in the time frame 12 to 24 hours post-partum is recommended. Furthermore, diagnosis should be performed not only due to temperature increase, but due to a combination of appropriate criteria such as clinical mammary gland changes, diminished milk production and reduced appetite [26]. Other parameters like cell count, established for cows' milk, are not common for diagnosis and data on thresholds are rare. For instance, a threshold of 5 × 10^6 ^cells per mL was proposed by Bertschinger and Bühlmann [27], while Persson et al. [28] suggested 10 × 10^6 ^cells per mL.

The procedure of milking sows in order to obtain samples is difficult and severely restricted by practical circumstances, but an absolutely needed prerequisite to obtain reliable results. Cleaning and disinfection are urgently required to eliminate skin flora or other contaminating microorganisms. Examinations on the skin flora on sows' teats do not exist, but studies on cows suggest, that staphylococci are the most common bacteria [29,30]. Pooled milking, as it is often used to get a sufficient amount of milk, most likely increases the risk of contamination. Because of this, and because of economic reasons, single teat samples from anterior and posterior glands were taken separately in our study, but pooled in the next laboratory step. In this way, the expected costs for bacteriological differentiation were reduced, while maintaining the hygienic demands. Moreover, the anatomy of the mammary gland can influence sampling: per teat, two complete gland systems end in two teat orifices without muscular sphincters [4]. Therefore, if only one gland system is affected by mastitis, the sampled milk might only consist of secretion from the other, healthy system, leading to false negative results.

Bacteria were found in all sows, but not necessarily in both anterior and posterior samples. Compared to similar studies, for instance Persson et al. [28] with no bacterial growth in 10% of all samples from agalactic and in 54% of all samples from healthy sows, this prevalence is high. However, in contrast to our project, no enrichment step was included in the bacteriological examination in that study, but samples were spread directly on blood agar. No selective agars were used, also lowering the probability to detect certain species. Moreover, in comparison to other studies [6,9-11], the documented incidence at farm level on the research farm was high with 48.2%. The general high incidence on farm level and the unknown background of that fact were reasons to realise this study. After antibiotic treatment in combination with non-steroidal anti-inflammatory drugs, administered after sampling, all sows recovered. One important fact regarding the actual presence of different bacteria species in sows' milk and the comparison with former investigations, conducted mainly in the 1980s and 1990s [31], is the use of appropriate methods for identification. Most isolated bacteria in our study were representatives of the families *Enterobacteriaceae*, *Staphylococcaceae*, *Streptococcaceae*, and *Enterococcaceae*. This spectrum is in agreement with other studies [15,32]; for instance, in a bacteriological examination of mammary gland changes in 663 sows suffering mastitis, mainly *E. coli *and *Klebsiella *spp. were detected, but also *Streptococci *and *Staphylococci *[22].

Like in other studies, the most commonly isolated bacteria from mastitis-affected sows belong to the class of coliforms, covering the bacterial genera *Escherichia*, *Klebsiella*, *Enterobacter *and *Citrobacter *[13,15,17]. Subsequently, the predominant role of coliform bacteria was clearly shown by Wegmann et al. [14]; in 131 complexes with mastitis, *E. coli *and *Klebsiella pneumonia *were isolated in 79%. This importance of *E. coli *has been confirmed in several studies [13,33,34] and in infection experiments, provoking clinical and haematological changes comparable to natural infections [35,36]. To emphasize the role of coliforms and to end the confusing terminology, the term 'coliform mastitis' was suggested for peripartal mastitis in sows [1].

Bacteria causing or at least accompanying the syndrome of coliform mastitis may originate from the intestinal flora of the sow, from the environment or from the oral flora of the neonatal piglet. The hypothesis of a galactogenous route of infection was corroborated by experiments carried out by Bertschinger et al. [15], in which a reduction of PPDS prevalence could be noticed after protection of the mammary gland against faecal contamination. Therefore, faecal contamination was postulated to be of paramount importance as a cause of puerperal mastitis. The faecal origin of *E. coli *isolated from sows' milk was also reported by Awad Masalmeh et al. [15]: in one quarter of 67 PPDS-affected sows O-serogroup-identical *E. coli *were detected in both milk and faecal samples. In another study comparing the bacterial flora of the uterus, the caecum, the ileum and the mammary gland, the prevalence of only gram-negative bacteria in the mammary glands and in the ileum of CM-affected sows was remarkable [23]. However, in this study, the faecal route of infection could not be confirmed due to the study design.

The lack of gram-negative bacterial culture growth in uterine samples supports the theory that uterine involvement in PPDS is of minor importance, as has been suggested in several studies [37-39]. However, infections of the urinary tract are strongly related to puerperal diseases, even though urinary infections are not apparent clinically [40]. In these infections as well, the most common organism associated with bacteriuria was found to be *E. coli *[41]. Therefore, not only faecal, but also urine contamination has to be considered as an infection source for PPDS. In this context, Bertschinger [1] suggested that the often recommended feed reduction might not act directly in the sow's organism, but indirectly through reduced exposure of the mammary complexes due to decreased amounts of faeces and urine contaminating the lying area. Up to now, information on the actual influences leading to the manifestation of mastitis in sows is widely lacking and further research is desirable.

## Conclusion

This study examines the prevalence of different bacteria in anterior and posterior glands in PPDS positive and negative sows. In conclusion, the bacterial flora of PPDS-affected and non-affected sows differs only slightly. Mainly ubiquitous bacteria were isolated and significant differences in the occurrence in anterior and posterior glands were not statistically confirmed. At the current state of knowledge, the reason for only some sows developing clinical signs of infection after contact with these ubiquitous bacteria remains unknown. The immune response and the actual outbreak of infection seems to depend on the immunological reactivity of the sow. Hence, one may hypothesise that developing clinical PPDS is largely dependant on the individual resistance of the sow and research is needed to define this individual resistance in detail.

## Competing interests

The authors declare that they have no competing interests.

## Authors' contributions

NK designed and coordinated the study, draft the manuscript and participated in the bacteriological analysis. IG carried out the bacteriological examinations and performed the statistical analysis. All authors read and approved the final manuscript.

## Authors' information

NK, Dr. med. vet, is Certified Veterinary Specialist in Microbiology (Fachtierärztin Mikrobiologie) and in Animal Hygiene (Fachtierärztin Tierhygiene) and leader of the researchgroup 'geMMA: structural and functional analysis of the genetic variation of the MMA-sydrome' (www.gemma-kiel.de). IG, MSc. Agr., is researcher in this group.
